# McArdle Disease: A Diagnostic Challenge Due to Nonspecific Clinical Manifestations

**DOI:** 10.7759/cureus.76010

**Published:** 2024-12-19

**Authors:** Sara Pereira, Paula Cerqueira, Sabina Azevedo, Marta Pereira, Selmira Faraldo

**Affiliations:** 1 Internal Medicine, Hospital Conde de Bertiandos, Unidade Local de Saúde do Alto Minho, Ponte de Lima, PRT; 2 Internal Medicine, Hospital Distrital de Santarém, Santarém, PRT

**Keywords:** glycogen storage disease type v, mcardle disease, myalgias, myophosphorylase deficiency, rhabdomyolysis

## Abstract

McArdle disease is a rare myopathy caused by hereditary myophosphorylase deficiency. It presents nonspecific symptoms, such as intolerance to physical exercise, early fatigue, and myalgias, and represents a paradigmatic example of one of the main challenges in clinical practice: the recognition of nonspecific and common symptoms as clinically relevant manifestations of rare diseases. The nonspecificity of symptoms leads to a frequent delay from the onset of first clinical signs to diagnosis. We present a case of McArdle disease diagnosed in a Portuguese adult patient. We believe this report will highlight the need to enhance knowledge and awareness about this rare pathology to diagnose and manage these patients promptly.

## Introduction

McArdle disease, or glycogen storage disease type V, is a rare autosomal recessive metabolic myopathy caused by a deficiency of the enzyme myophosphorylase. The reported prevalence of this disease ranges from 1:100,000 to 1:350,000. However, a study based on next-generation sequencing data suggests that the six mutations most frequently associated with McArdle disease are present in a significantly larger number of individuals (approximately one in 7,650) [1]. The clinical manifestations of McArdle disease - such as exercise intolerance, muscle weakness, early fatigue, myalgias, and muscle cramps - are nonspecific and may present from childhood onwards. These nonspecific symptoms make diagnosis challenging and often result in diagnostic delays, with the median age at diagnosis being 33 years [2,3]. In Portugal, very few cases of McArdle disease diagnosed in adulthood have been reported in the literature [4,5]. We discuss a case of McArdle disease diagnosed in adulthood, highlighting the need to increase awareness and understanding of this rare condition to enable earlier diagnosis and management of affected individuals.

## Case presentation

The patient was a 35-year-old male with a medical history of dyslipidemia, overweight (BMI of 27.8 kg/m²), and obstructive sleep apnea syndrome. He had been on daily atorvastatin 20 mg for dyslipidemia management for the past five months. He was also on nocturnal non-invasive ventilation. He reported a family history (one sister) of myopathy, though the formal diagnosis was unknown to him. Their parents were non-consanguineous. He presented to the emergency department with fatigue on minimal exertion, myalgias, and gait claudication that had persisted for at least 10 years but markedly worsened in the past four months. He reported that previous episodes had been underestimated, including by himself. Despite not engaging in sports, his work involved walking approximately 6 km daily and performing anaerobic physical tasks. Symptoms persisted stably but without improvement throughout physical activity, but resolved with rest. He denied other complaints, toxic substance use, or recent infections.

A physical examination of the patient revealed no abnormalities. Initial bloodwork (Table 1) showed elevated myoglobin (601 ng/mL) and creatine kinase (CK 5800 U/L) levels. Serum creatinine (0.71 mg/dL) and liver biology enzymes (alanine aminotransferase, aspartate aminotransferase, alkaline phosphatase, and gamma-glutamyl transferase) were within normal ranges. He was diagnosed with statin-induced rhabdomyolysis and was discharged with instructions to discontinue atorvastatin, refrain from physical activity, and increase oral hydration.

**Table 1:** Summary of clinical investigation

Laboratory parameter	Result (emergency department)	Result (reassessment in the outpatient clinic)	Normal range
Hemoglobin	15.2 g/dL	15.6 g/dL	12-16 g/dL
Leukocyte count	5.32 x10^9^/L	5.63 x10^9^/L	4.0-11.0 x10^9^/L
Platelet count	160 x10^9^/L	160 x10^9^/L	150-400 x10^9^/L
Urea	33 mg/dL	36 mg/dL	<50 mg/dL
Creatinine	0.71 mg/dL	1.0 mg/dL	0.6-1.1 mg/dL
Sodium	139 mEq/L	140 mEq/L	135-145 mEq/L
Potassium	4.5 mEq/L	4.6 mEq/L	3.5-5.0 mEq/L
Creatine kinase	5800 U/L	1468 1U/L	<190 U/L
Myoglobin	601 ng/mL	600 ng/mL	10-95 ng/mL
Aspartate aminotransferase	29 U/L	102 U/L	<35 U/L
Alanine aminotransferase	24 U/L	103 U/L	<33 U/L
Gamma-glutamyl transferase	32 U/L	32 U/L	7-32 U/L
Alkaline phosphatase	45 U/L	52 U/L	35-105 U/L
Total bilirubin	0.9 mg/dL	0.8 mg/dL	<1.2 mg/dL
Triglycerides	-	339 mg/dL	<150 mg/dL
Total cholesterol	-	301 mg/dL	<190 mg/dL
LDL cholesterol	-	212 mg/dL	<130 mg/dL
HDL cholesterol	-	42 mg/dL	>40 mg/dL

After two weeks, during which he adhered to the given recommendations, the patient was reassessed in the outpatient clinic. His complaints and physical examination findings were unchanged and consistent with prior evaluations. Gowers' sign was negative, not suggesting weakness of the proximal muscles. Laboratory analysis revealed persistent elevations in serum myoglobin (600 ng/mL) and CK (14681 U/L) and newly-elevated alanine aminotransferase (103 U/L) and aspartate aminotransferase (102 U/L), while serum creatinine was within reference ranges. Dyslipidemia was confirmed, with total cholesterol at 301 mg/dL, LDL cholesterol at 212 mg/dL, HDL cholesterol at 42 mg/dL, and triglycerides at 339 mg/dL. Bloodwork results are summarized in Table 1. Urine analysis was normal.

The patient was admitted for further evaluation and started on intravenous fluid therapy. His family history of unspecified myopathy, associated with the persistence of clinical and laboratory abnormalities despite statin discontinuation, heightened the suspicion of a hereditary myopathy and raised doubts about the possibility of statin-induced rhabdomyolysis as the sole etiology. Genetic testing (multigene panel test) identified the p.(Arg50*) variant in homozygosity in the PYGM gene, located at genomic coordinates chr11:64527223G>A (GRCh37). This variant corresponds to transcript NM_005609 and confirms the diagnosis of McArdle disease.

The patient did not develop acute kidney injury, hyperkalemia, myoglobinuria, hyperuricemia, or hyperlactatemia. The increase in serum transaminases, most likely of muscular origin, was resolved without any specific measures. Electromyography (EMG) showed no signs of residual myopathic damage, recent inflammatory myopathy, or sensory/motor polyneuropathy. He was evaluated by Neurology, which contributed to therapeutic guidance and outpatient follow-up planning. Progressive clinical and laboratory improvement was observed during hospitalization, with CK levels decreasing to 1076 U/L at discharge after 10 days.

The patient was referred for evaluation by Neurology, Nutrition, and Genetic Counseling teams. He was advised to engage in regular low-to-moderate intensity aerobic exercise while avoiding strenuous efforts and anaerobic activity. Recommendations were also made regarding diet and hydration, emphasizing a carbohydrate-rich diet, including simple carbohydrates consumed shortly before physical activity, and adequate hydration. Dietary measures aimed at improving dyslipidemia were suggested, resulting in progressive improvement: around 12 months later, his lipid profile showed total cholesterol of 218 mg/dL, LDL cholesterol of 173 mg/dL, HDL cholesterol of 31 mg/dL, and triglycerides of 177 mg/dL. The patient also achieved significant weight loss, attaining a normal BMI (24.7 kg/m²). It was decided to keep the patient off lipid-lowering medications.

## Discussion

Fatigue and myalgia are nonspecific complaints that are often underestimated. When associated with rhabdomyolysis in a patient on statin therapy, drug-induced myopathy is one possible cause, but not the only one - as demonstrated in this clinical case. McArdle disease, or glycogen storage disease type V, is a rare metabolic myopathy caused by mutations in the PYGM gene. This gene encodes glycogen myophosphorylase, an enzyme essential for breaking down glycogen into glucose-1-phosphate in skeletal muscle. Deficiency of this enzyme prevents the utilization of glycogen as an energy source during physical activity, leading to its intracellular accumulation and impaired muscle contraction [6]. 

Characteristic clinical manifestations include exercise intolerance, muscle weakness, early fatigue, myalgias, and muscle cramps, which may occur just seconds or minutes after initiating physical activity. The clinical case described here illustrates these classic but nonspecific features of McArdle disease, which can be triggered by physical activity of varying intensity. In this case, the patient developed symptoms and rhabdomyolysis following only moderate-intensity physical activity. In McArdle disease, the second wind phenomenon is often observed. It consists of a marked improvement in physical capacity and tolerance several minutes after an initial period of fatigue. This improvement is due to the mobilization and metabolism of alternative energy sources (fatty acids and serum glucose) and increased muscle blood flow [7].

While characteristic and common, this phenomenon may occasionally be absent, as observed in this case. Physical examination findings, such as muscle atrophy of the shoulder girdle and paravertebral muscles or ptosis, are rarely observed in McArdle disease [2,8]. McArdle disease is almost universally associated with rhabdomyolysis. Even at rest, patients may exhibit elevated serum CK and myoglobin levels. Rhabdomyolysis often worsens significantly with physical activity/exercise or in the presence of other stressors (e.g., statin use), leading to elevated serum CK and myoglobin levels, myoglobinuria, and potential progression to acute kidney injury, hyperkalemia, and metabolic acidosis. Approximately 50% of patients experience episodes of rhabdomyolysis and myoglobinuria induced by physical activity [9]. 

The diagnosis of McArdle disease can be confirmed via genetic testing, identifying pathogenic mutations in the PYGM gene, or through muscle biopsy, which demonstrates absent myophosphorylase activity. Muscle histology characteristically reveals subsarcolemmal glycogen deposits with normal structure [10]. Advances in diagnostic tools, particularly genetic testing, have facilitated the identification of an increasing number of PYGM gene mutations associated with McArdle disease. In the presented clinical case, the definitive diagnosis was established by identifying one such mutation. Given the current availability and efficacy of genetic testing panels for metabolic myopathy-associated mutations, muscle biopsy is reserved for cases where genetic testing is unavailable or when its results are inconclusive. 

Additional laboratory findings may include a lack of serum lactate elevation during an anaerobic exercise test and/or a marked increase in serum ammonia levels in response to exercise. Furthermore, de Luna et al. propose that assessing leukocyte myophosphorylase expression may serve as a useful, non-invasive diagnostic tool for McArdle disease [11], particularly when novel or clinically indeterminate genetic variants are identified. MRI may reveal muscle atrophy and fat replacement, primarily in the paraspinal and shoulder girdle muscles [12]. Other diagnostic tests can suggest or support the diagnosis. Patients may exhibit persistently elevated serum CK levels, even at rest [8], as observed in this case. During anaerobic exercise, EMG demonstrates a lack of electrical activity in contracting muscles, whereas, at rest, it may be normal or show nonspecific changes, as in the described case. 

McArdle disease itself does not reduce the life expectancy of affected individuals [2], but it is associated with a diminished quality of life. Currently, no specific treatment exists for McArdle disease. Gene therapy is an area of ​​interest, but there are currently no approved genetic treatments for this condition. To minimize symptoms, the risk of rhabdomyolysis, and its complications, the management involves symptomatic treatment, appropriate regulation of physical activity, and dietary and hydration measures. A carbohydrate-rich diet may alleviate symptoms by maintaining hepatic glycogen stores and enabling the subsequent mobilization of hepatic glucose during physical activity [13,14].

The consumption of simple carbohydrates such as sucrose (e.g., in sports drinks) before exercise can enhance exercise tolerance, particularly during aerobic activities [15]. However, the consumption of other fast-acting simple carbohydrates, such as glucose and fructose, does not appear to provide similar benefits [14]. Engaging in regular low-to-moderate intensity aerobic exercise, such as walking or cycling, has demonstrated benefits in improving exercise tolerance [16]. Controlled and consistent aerobic activity fosters the development of mitochondrial oxidative capacity within muscle cells, leading to increased exercise tolerance, improved ability to achieve the second wind phenomenon, enhanced endurance of muscle fibers with a reduced risk of myoglobinuria and rhabdomyolysis, and lower serum CK levels [17,18].

The management of dyslipidemia in these patients might be challenging. Statins should be used cautiously, with fluvastatin, pravastatin, or pitavastatin being preferable due to their lower myopathy risk [19]. PCSK9 inhibitors appear to be effective and safe in this context and may be an appropriate option for patients with muscle disorders or statin contraindications [20].

## Conclusions

While fatigue and myalgia are nonspecific complaints that are often underestimated, McArdle disease is a rare metabolic myopathy with nonspecific symptoms often mistaken for physical deconditioning. Consequently, its diagnosis is frequently delayed, as exemplified in the presented case. To accurately and promptly diagnose McArdle disease, clinicians face one of the primary challenges in medical practice: recognizing common and nonspecific symptoms as clinically significant manifestations of rare diseases. This report highlights the importance of raising awareness and enhancing knowledge about this condition among healthcare professionals - including physicians, nurses, physiotherapists, and nutritionists - to ensure early diagnosis and management, thereby improving these patients’ quality of life.
